# Speed breeding for multiple quantitative traits in durum wheat

**DOI:** 10.1186/s13007-018-0302-y

**Published:** 2018-05-14

**Authors:** Samir Alahmad, Eric Dinglasan, Kung Ming Leung, Adnan Riaz, Nora Derbal, Kai P. Voss-Fels, Jason A. Able, Filippo M. Bassi, Jack Christopher, Lee T. Hickey

**Affiliations:** 1 Queensland Alliance for Agriculture and Food Innovation, The University of Queensland, St Lucia, Brisbane, QLD 4072 Australia; 2 Department of Ecology and Environmental Engineering, The University of 8 Mai 1945, 24000 Guelma, Algeria; 3 School of Agriculture, Food and Wine, Waite Research Institute, The University of Adelaide, Urrbrae, Adelaide, SA 5064 Australia; 4 International Center for the Agricultural Research in the Dry Areas, 10000 Rabat, Morocco; 5 Queensland Alliance for Agriculture and Food Innovation, The University of Queensland, Leslie Research Facility, Toowoomba, 4350 QLD Australia

**Keywords:** Root architecture, Crown rot, Leaf rust, Drought adaptation, Segregating populations, *Fusarium*, Speed breeding, Trait pyramiding

## Abstract

**Background:**

Plant breeding requires numerous generations to be cycled and evaluated before an improved cultivar is released. This lengthy process is required to introduce and test multiple traits of interest. However, a technology for rapid generation advance named ‘speed breeding’ was successfully deployed in bread wheat (*Triticum aestivum* L.) to achieve six generations per year while imposing phenotypic selection for foliar disease resistance and grain dormancy. Here, for the first time the deployment of this methodology is presented in durum wheat (*Triticum durum* Desf.) by integrating selection for key traits, including above and below ground traits on the same set of plants. This involved phenotyping for seminal root angle (RA), seminal root number (RN), tolerance to crown rot (CR), resistance to leaf rust (LR) and plant height (PH). In durum wheat, these traits are desirable in environments where yield is limited by in-season rainfall with the occurrence of CR and epidemics of LR. To evaluate this multi-trait screening approach, we applied selection to a large segregating F_2_ population (n = 1000) derived from a bi-parental cross (Outrob4/Caparoi). A weighted selection index (SI) was developed and applied. The gain for each trait was determined by evaluating F_3_ progeny derived from 100 ‘selected’ and 100 ‘unselected’ F_2_ individuals.

**Results:**

Transgressive segregation was observed for all assayed traits in the Outrob4/Caparoi F_2_ population. Application of the SI successfully shifted the population mean for four traits, as determined by a significant mean difference between ‘selected’ and ‘unselected’ F_3_ families for CR tolerance, LR resistance, RA and RN. No significant shift for PH was observed.

**Conclusions:**

The novel multi-trait phenotyping method presents a useful tool for rapid selection of early filial generations or for the characterization of fixed lines out-of-season. Further, it offers efficient use of resources by assaying multiple traits on the same set of plants. Results suggest that when performed in parallel with speed breeding in early generations, selection will enrich recombinant inbred lines with desirable alleles and will reduce the length and number of years required to combine these traits in elite breeding populations and therefore cultivars.

**Electronic supplementary material:**

The online version of this article (10.1186/s13007-018-0302-y) contains supplementary material, which is available to authorized users.

## Background

Durum wheat (*Triticum durum* Desf.) is one of the major staple crops in the Mediterranean region. It is known for its unique properties such as hardness, yellow pigment and high protein content. Therefore, it is ideal for making pasta [1–4], couscous and burghul [2]. However, durum wheat production continues to face many challenges associated with environmental constraints, pests and diseases [5, 6]. The number one constraint limiting production is insufficient water availability, as variable in-season rainfall can dramatically affect yield and grain quality [1]. In addition, durum wheat production is restricted due to susceptibility to several fungal diseases. In comparison to bread wheat (*Triticum aestivum* L.), durum wheat cultivars are particularly susceptible to *Fusarium* species, including *Fusarium* *graminearum* (*Fg*) and *Fusarium pseudograminearum* (*Fp*), which cause fusarium head blight [7, 8] and crown rot (CR) [9–11], respectively. CR is an increasing issue in many parts of the world due to adoption of minimum tillage practices, which retains the inoculum on stubble across seasons [12, 13]. Notably, yield losses due to CR are exacerbated under terminal drought conditions [14–18], thus drought adaptation features related to increased water uptake and or water-use efficiency are expected to reduce production losses in CR affected production systems [19]. Air-borne pathogens, such as rust, also pose an ongoing constraint as races constantly evolve to acquire new virulence against the deployed resistance genes. Several studies have reported a number of highly virulent isolates of the leaf rust pathogen *Puccinia triticina*, causing leaf rust (LR), in major durum production areas such as North Africa [20], Southern Europe [21, 22], and West Asia [23] and have now rendered susceptible many previously resistant durum cultivars.

Traditional cereal breeding programs around the world have delivered many significant improved varieties over the past 100 years. Nonetheless, progress is slow, in part due to lengthy breeding cycles which often takes 10–15 years from cross to cultivar release [24]. A major challenge is combining large numbers of traits that are polygenic in nature [25]. While marker-assisted selection (MAS) has proven itself a useful tool in crop improvement programs, the approach is most effective when targeting a small number of genes with large effect, such as leaf rust resistance genes (e.g. *Lr23*) in bread and durum wheat [26] and *Yr15* in durum wheat [27]. In addition, MAS can only be applied if the target gene or quantitative trait loci (QTL) responsible for the trait of interest is known. Thus, MAS is less feasible for complex traits for which little is known about the underlying genetic controls [28]. Recently, genomic selection (GS) has overcome the limitations of MAS as it uses genome-wide markers to estimate the breeding values (EBVs), which provide an estimate of the genomic merit associated with all minor or major effects across the entire genome [29, 30]. GS also facilitates selection for multiple traits in parallel; yet despite the efficiency and promise of this breeding tool, costs associated with genotyping large numbers of selection candidates is still relatively high to facilitate full adoption in the majority of wheat breeding programs. Further, GS is typically applied to inbred lines [31–33], therefore the rate of progress is limited by the time required to make crosses and generate new selection candidates that are genetically stable.

A technology that permits rapid generation advancement, named ‘speed breeding’, has been refined to achieve up to 6 generations of wheat per year [34], thus presenting a useful tool to reduce the length of breeding cycles. Several phenotyping protocols adapted to the speed breeding system have been developed, which enable characterisation and selection for important traits. Examples include seminal root traits for drought adaptation [35], grain dormancy for tolerance to pre-harvest sprouting [36, 37], and disease traits such as adult plant resistance (APR) to LR [38], stripe rust [39] and yellow spot [40] in bread wheat.

Importantly, these protocols provide phenotypes that correspond with field-based measures [37, 38, 40, 41]. While these reported methods focus on a single trait, there is an opportunity to integrate phenotyping and selection for multiple traits in the same plant generation grown under speed breeding conditions.

In this study, we designed and applied a novel multi-trait phenotyping method adapted to speed breeding for characterising fixed lines out of season and provide selection pressure during early generations of durum wheat. To test the effectiveness of early generation selection, we applied selection to a large F_2_ population for multiple traits in order to evaluate the shift in phenotypic response and discuss the opportunity to accelerate pyramiding of multiple target traits in durum breeding populations.

## Methods

### Plant materials

A bi-parental population was generated to combine multiple desired traits. Parents consisted of an elite ICARDA durum line, Outrob4 (Ouassel–1/4/GdoVZ512/Cit//Ruff/Fg/3/Pin/Gre//Trob) and the Australian durum cultivar Caparoi (LY 2.6.3/930054). The ICARDA line was selected for its desirable tolerance to severe drought conditions, as well as its lack of yield losses when grown under severe CR infection with a response of moderately resistant to moderately susceptible (MRMS) in Latakia, Syria. Caparoi is a high quality durum cultivar that is very susceptible to CR and displays a moderately resistant and resistant (MRR) response to LR. The two parental lines and two standards, depending on the traits to be measured, were included in 12 replicates in all experiments using a randomised complete block design (RCBD). Standards included spring bread wheats: Mace (wide root angle), Scout (narrow root angle), Thatcher (susceptible to LR), Thatcher + *Lr34* (adult plant resistance to LR), Sunguard (moderately resistant to CR) and durum wheat Yawa (very high yielding, susceptible to CR). Details for all standards and parental lines are provided in Table 1.

**Table 1:** Trait means and standard error (SE) for parents and standards evaluated in the multi-trait screening of F_2_ and F_3_ experiments

Trait	Genotype	Pedigree	Standard/parents	F_2_		F_3_	
				Mean	SE	Mean	SE
Root angle	Mace	Wyalkatchem/Stylet//Wyalkatchem	Wide	79.6	4.3	67.0	6.3
	Scout	Sunstate/Qh71-6//Yitpi	Narrow	31.9	5.1	45.3	7.1
	Outrob4	Ouassel-1/4/Gdovz 512/Cit//Ruff/Fg/3/Pin/Gre//Trob, syn.Fadda98	Parent 1	50.1	2.1	32.4	6.2
	Caparoi	LY 2.6.3/930054	Parent 2	77.3	6.6	55.1	6.3
Root number	Mace	–	High	4.6	0.2	3.1	0.3
	Scout	–	Low	3.2	0.5	2.7	0.3
	Outrob4	–	Parent 1	4.3	0.4	4.7	0.3
	Caparoi	–	Parent 2	3.7	0.3	3.5	0.3
Crown rot	Sunguard	Sun289e/Sr2janz	Resistant	3.8	0.4	3.8	0.6
	Yawa	Westonia/Kalka//Kalka/Tamaroi///Rac875/Kalka//Tamaroi	Susceptible	6.3	0.4	6.2	0.6
	Outrob4	–	Parent 1	4.3	0.3	4.7	0.6
	Caparoi	–	Parent 2	7.5	0.4	6.8	0.6
Leaf rust	Thatcher + *Lr34*	Thatcher*6/PI-58548	Resistant	5.5	0.3	6.9	0.6
	Thatcher	Marquis/Iumillo durum//Marquis/Kanred	Susceptible	8.6	0.3	9.0	0.6
	Outrob4	–	Parent 1	5.4	0.5	6.4	0.6
	Caparoi	–	Parent 2	3.1	0.2	2.0	0.7
Plant height	Mace	–	Short	46.0	2.2	54.1	3.7
	Thatcher	–	Tall	62.9	3.5	77.9	3.7
	Outrob4	–	Parent 1	56.2	3.9	61.0	3.7
	Caparoi	–	Parent 2	49.0	2.0	50.2	4.1

### Crossing, population development and selection

**Fig. 1:**
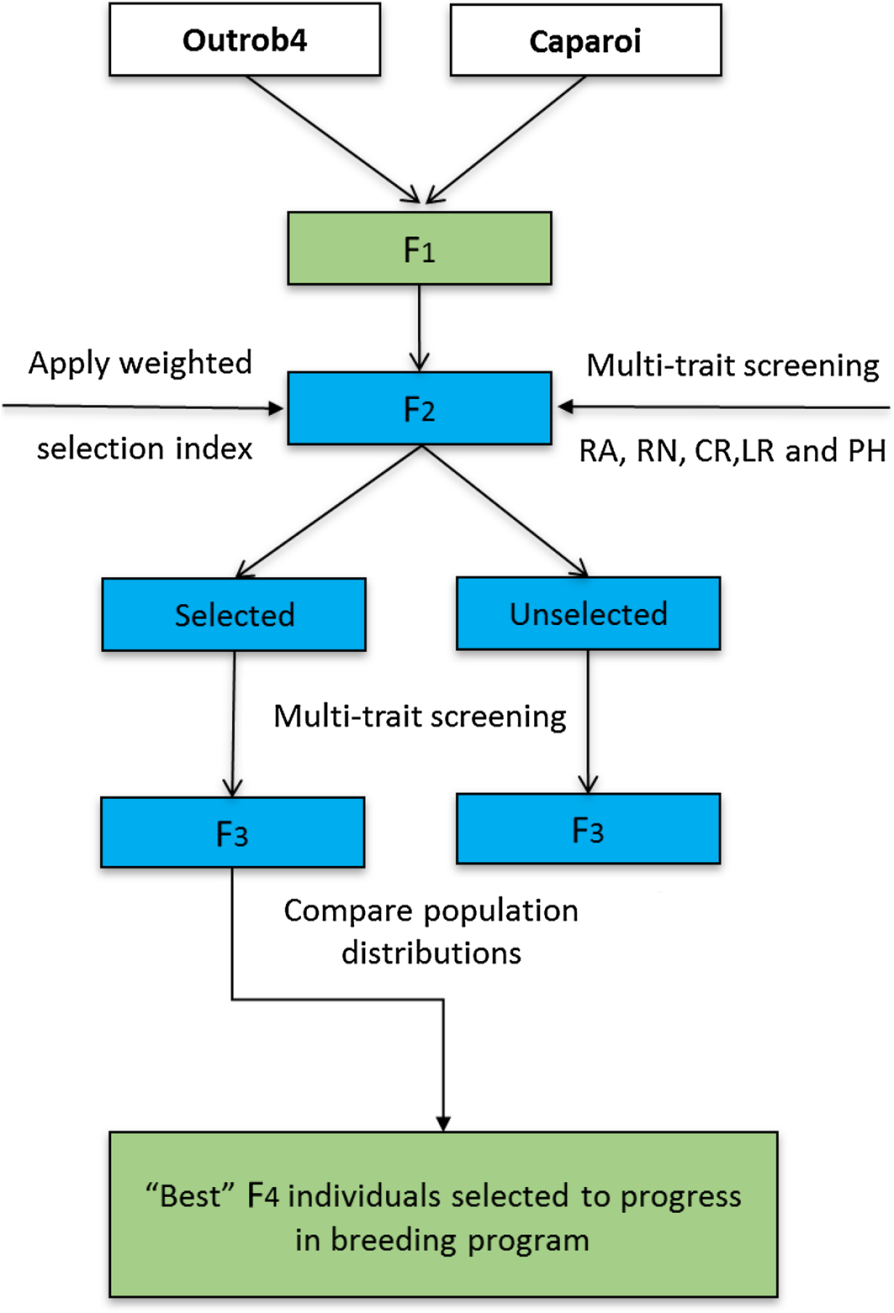
The breeding strategy for applying selection in early segregating generations to reach superior inbreds in a period of 11 months. The figure highlights the crossing parents and further generations where a weighted SI was used. The blue colour indicates generations where the phenotyping was conducted. The green coloured generations indicate the generations subject to growth under speed breeding for the entire cycle without selection

An overview of the population development and multi-trait screening applied in this study is illustrated in Fig. 1. Outrob4 and Caparoi were grown in the speed breeding system to rapidly bring them to the flowering stage for crossing. Outrob4 was used as the female and Caparoi as the pollen donor. Approximately 9 weeks after sowing, the F_1_ seed from physiologically mature spikes were harvested and placed in an air-forced dehydrator at 35° for 5 days, and subsequently threshed by hand. 1000 of the resulting F_2_ seeds were sown and phenotyped as indicated below. Selection was applied using a weighted selection index (SI) incorporating all phenotypic data. The 100 best performing individuals were ‘selected’ along with 100 random individuals, which represented the ‘unselected’ population. To investigate the response to selection, the selected and unselected sets were phenotyped following the same procedure using a RCBD design with five individuals representing each F_3_ family (total 1000 plants) and 12 replicates per standard.

### Multi-trait phenotyping procedure for F_2_ and F_3_

**Fig. 2:**
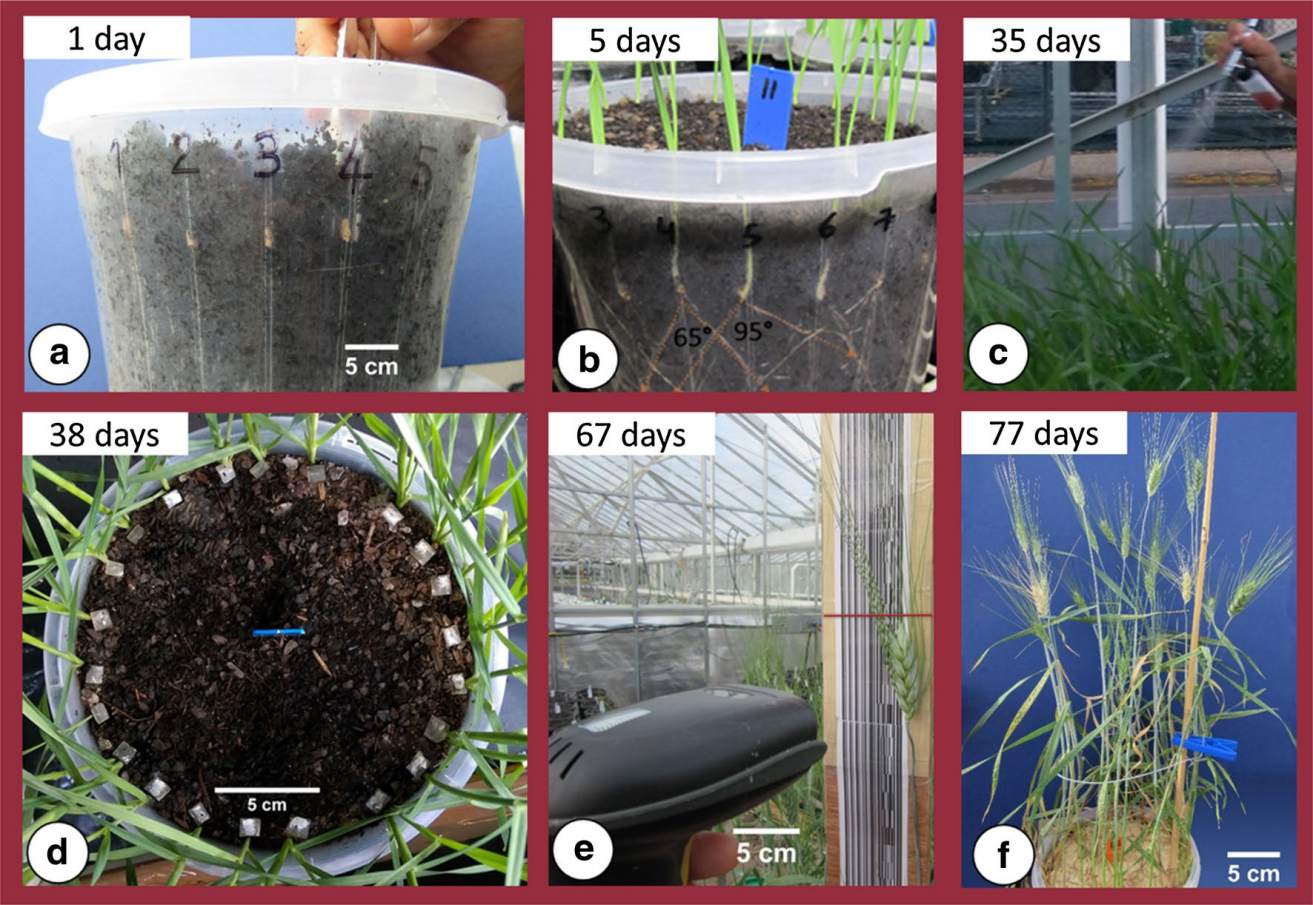
Visual summary of a generation from sowing to harvest using the multi-trait phenotyping procedure: **a** seeds sown in the clear-pot, **b** seminal root image analysis, **c** plants inoculated with leaf rust using airbrush method, **d** plants inoculated with Fusarium crown rot, **e** plant height measured using a barcode reader, and **f** whole-pot view at the time of crown rot assessment during the grain filling stage

The F_2_ and F_3_ generations were subject to selection for CR, RA, RN, LR and PH by adapting the ‘clear pot’ method reported by Richard et al. [35] (Fig. 2a). The phenotyping method described below was performed under speed breeding conditions where each generation was completed within 77 days from sowing to harvest.

### Sowing and root phenotyping

Clear (transparent) pots (ANOVApot^®^, 200 mm diameter, 190 mm height) were filled with composted fine pine bark (70%) particles (0–5 mm) pre-mixed with coco peat (30%) to increase water holding capacity of the medium. To reduce the acidity of the medium for growing durum wheat, Dolomite was added at a rate of 1 kg per 1 m^3^ of soil providing a pH of 6.5. The F_2_ seeds were sown according to the Richard et al. [35] clear pot method, using the RCBD design where 24 seeds were sown in each 4 L pot, which enables evaluation of up to 600 plants/m^2^ of glasshouse space (Fig. 2a). Seeds were sown using tweezers to carefully position the embryo down and facing the wall of the pot to allow good visibility of the seminal roots following germination. Plants were grown in the glasshouse under diurnal natural light conditions adopting a constant temperature (17 ± 2 °C) as recommended [35]. Images were captured at two time points: 5 days (seminal roots 3–5 cm in length) for RA and 10 days after sowing (DAS) for RN, using a Canon PowerShot SX600 HS 16MP Ultra–Zoom Digital camera. The first set of images were analysed for seminal root angle (Fig. 2b), where the angle between the first pair of seminal roots emerging from the seed was measured using ImageJ (http://imagej.nih.gov/ij/). Images captured to determine the number of seminal roots were used to score visible roots through the transparent wall. Following the final image capture at 10 DAS, growth conditions were changed to a ‘speed breeding’ regime using constant light and controlled temperature of 22/17 °C (day/night) to accelerate plant development. Under such conditions, plants obtain the adult growth stage (i.e. stem elongation) within about 3 weeks [38]. At this time, approximately 5 g of slow release Osmocote^®^ fertiliser was added to each pot to provide adequate nutrients to sustain healthy plant growth.

### Phenotyping leaf rust response

At 35 DAS, plants were inoculated with LR spores. By this time most plants had reached the stem elongation growth stage (Zadoks GS 39), which is essential to attain infection types that resemble adult plants in the field [38, 39]. The *Puccinia triticina* (*Pt*) pathotype 104–1, 2, 3, (6), (7), 11, 13 was used for population screening. This *Pt* isolate was first reported in South Australia in 2000 [42] and is virulent for *Lr1, Lr3a, Lr14a, Lr16, Lr17a, Lr20, Lr24 and Lr27* resistance genes. This *Pt* isolate was used due to the absence of durum specific *Pt* pathotypes in Australia and since LR disease on durum wheat crops in Australia is caused by bread wheat pathotypes [43].

*Pt* urediniospores were suspended in light mineral oil (Isopar 6) at a rate of 0.005 g mL^−1^. The inoculum was applied using the airbrush method (Fig. 2c), as reported by Riaz et al. [38]. Plants were then lightly misted with deionized water and placed overnight in a dew chamber with 100% humidity using an ultrasonic fogger. Post–inoculation, plants were grown under diurnal and controlled temperatures of 17/22 °C (night/day). These conditions favour pathogen development and provide significant differentiation between resistant and susceptible genotypes. Each plant was evaluated 49 DAS (i.e. 14 days after infection) for resistance using a 0–9 scale, where 0 is resistant and 9 is very susceptible [44].

### Phenotyping for crown rot response

At 38 DAS, 3 days after LR inoculation, plants were inoculated with *F. pseudograminearum*. The isolate of *F. pseudograminearum* used in this experiment was derived from CR infected wheat plants collected from a farmer’s field located at Brookstead (Queensland, Australia), thus named “BE”. This isolate was tested with eight other isolates collected from different wheat fields located in the eastern wheat-belt of Australia (Queensland and New South Wales) and BE was selected as the most aggressive isolate causing symptoms on durum wheat and barley cultivars (Additional file 1: Table S1). Prior to inoculation, the isolate was cultured on 20% V8 medium comprising 200 mL of V8 juice, 2 g of CaCO3 and 20 g of agar, which was mixed in 800 mL of distilled water and pH adjusted to 7.2 [45]. The mixture was then autoclaved for 30 min at 121 °C. The medium was poured into Petri dishes and left to solidify. The BE isolate was cultured on Petri dishes and left at room temperature (20–25 °C) for 2 weeks to generate sufficient mycelial growth and conidial spores for inoculation. For each screening experiment, a pure source of the isolate was used to avoid changes in pathogenicity of the isolate, which can occur as a result of repeated media culturing without a host.

To reduce variation in the timing of infection among plants, a piece of cultured F. *pseudograminearum* (1 cm^2^) was placed at the base of the stem of each plant using tweezers (Fig. 2d). The soil surface was then covered with ground sterilised millet. Moisture at the surface was maintained by lightly spraying demineralised water three times per day for the first week to encourage mycelium growth and infection. Within 5 days, the surface of the pot was covered with white to pink coloured mycelium, which facilitated consistent infection at the base of the stem. Twenty-five days post-inoculation (63 DAS), disease severity for each plant was assessed visually by scoring the level of discoloration of the base of the stem using a 0–9 scale, where 0 is resistant and 9 is very susceptible.

### Plant height

A bluetooth barcode reader (Laser Bar Code Scanner) was used to measure height for each plant at maturity, 9 weeks after sowing (Fig. 2e). Barcode readings were at 1 cm intervals and the records were connected to a tablet allowing for instant data collection.

### Data analyses

The phenotypic value for each trait (RA, RN, CR, LR and PH) for each F_2_ plant was used to generate population distributions using GraphPad prism version 6 (Graphpad Software Inc). The mean for standards and parental lines were calculated for the F_2_ and F_3_ screens. The confidence interval (95%) was calculated using MS Excel for the mean of replicated parental lines included in the F_2_ screen.

F_2_ individuals were deemed to display transgressive segregation if phenotype scores could be identified outside the confidence interval of parental lines. A SI incorporating information for all traits was calculated according to Crosbie et al. [46] where the prefered traits included: narrow RA, high RN, tolerance to CR, resistance to LR and short PH. The following weights were applied based on the importance of each trait [47] in The University of Queensland durum wheat pre-breeding program: 35% for crown rot, 30% for root angle, 15% for LR, 10% for root number, 10% for PH. As the highest priority was resistance to CR, low weighting was applied to PH. CR resistance is typically correlated with a reduction in PH in wheat and barley [48–53], however high infection can retard plant growth, which further complicates selection for this trait. The SI was used to rank the 817 F_2_ individuals which had data for all traits. Individuals with a minimum of one ‘NA’ value were excluded from selection. The top 100 performing F_2_ individuals were considered the ‘selected’ set.

Best linear unbiased predictors (BLUPs) were calculated for each F_3_ family in the selected and unselected sets, plus parents and standards. BLUPs were calculated by fitting a linear model in ASReml-R [54], where genotype, replicate and pot were fitted as random terms in the model. The broad-sense heritability was calculated using the predicted variance components which were calculated using residual maximum likelihood, as described by Cullis et al. [55]. In the F_2_ and F_3_ experiments, the broad-sense heritability was calculated using repeated measures for inbred lines (i.e. parental lines and standards).

BLUPs were used for comparison to perform selection on the basis of genetic merit of each individual using the phenotypic response. Analysis of variance was performed for the F_3_ families to determine whether the selected set was significantly different in comparison to the unselected set. Analyses were performed using ASReml in R [54]. Selection was repeated for the F_3_ selected set using the same SI and weightings detailed above. The top 10% of best performers in the population were retained and rapidly advanced via single seed descent in the speed breeding system to develop inbred lines.

## Results

### Phenotypes displayed by standards

Using multi-trait phenotyping, above and below ground traits including adaptive root traits seminal RA and RN, and economically significant diseases CR and LR were screened (Fig. 2f). The standards performed as expected even though the absolute values for each trait varied across the F_2_ and F_3_ experiments. For example, the standard for wide RA (Mace) consistently displayed a wider mean RA than the narrow standard (Scout) in the F_2_ (79.6° vs. 31.9°) and F_3_ (67° vs. 45.3°) screening experiments (Table 1). Mace, a bread wheat cultivar grown across Australia, displayed not only the widest RA, but also the highest RN in the F_2_ experiment (4.6), but was lower in the F_3_ experiment (3.1). On the other hand, Scout displayed a lower RN in both the F_2_ (3.2) and F_3_ (2.7) screening experiments.

**Fig. 3:**
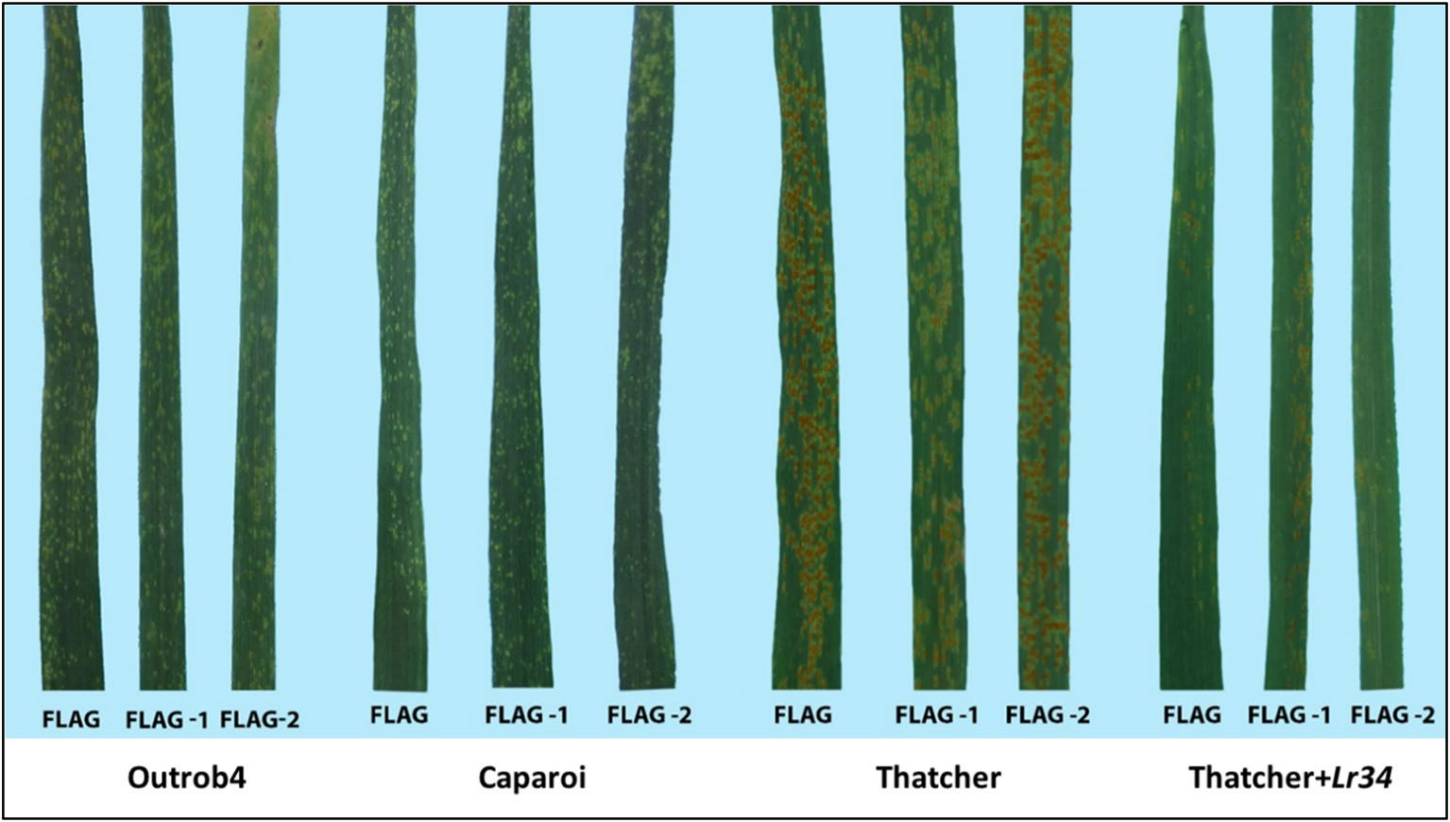
Leaf rust response on the flag leaf for parental genotypes (Outrob4 and Caparoi) and standards (Thatcher and Thatcher + Lr34)

The bread wheat standards for CR displayed very consistent phenotypes across the two experiments (Table 1). As expected, the incidence of CR was lower in Sunguard (3.8), which is considered moderately resistant (MR). In the field, the cultivar Sunguard is rated (MRMS) to CR (GRDC-NVT 2016). On the other hand, Yawa (an Australian durum wheat) was used as a susceptible standard for CR and displayed a mean score of 6.3 and 6.2 across both experiments. Thatcher was included as a very susceptible (VS) standard for LR, and as expected, allowed the pathogen to freely produce large pustules and spore masses (Fig. 3). Thatcher attained susceptible means of 8.6 and 9.0 in F_2_ and F_3_ experiments, respectively. In contrast, the standard for APR to LR (Thatcher + *Lr34*), showed a moderate level of resistance with a mean score of 5.5 in the F_2_ experiment and a lower level of resistance in the F_3_ experiment 6.9. The standards for PH were Mace for short height (46, 54.1 cm) and Thatcher for the tall types (62.9, 77.9 cm) in the F_2_ and F_3_ experiments, respectively.

### F_2_ screening and population distributions

#### Root angle

**Fig. 4:**
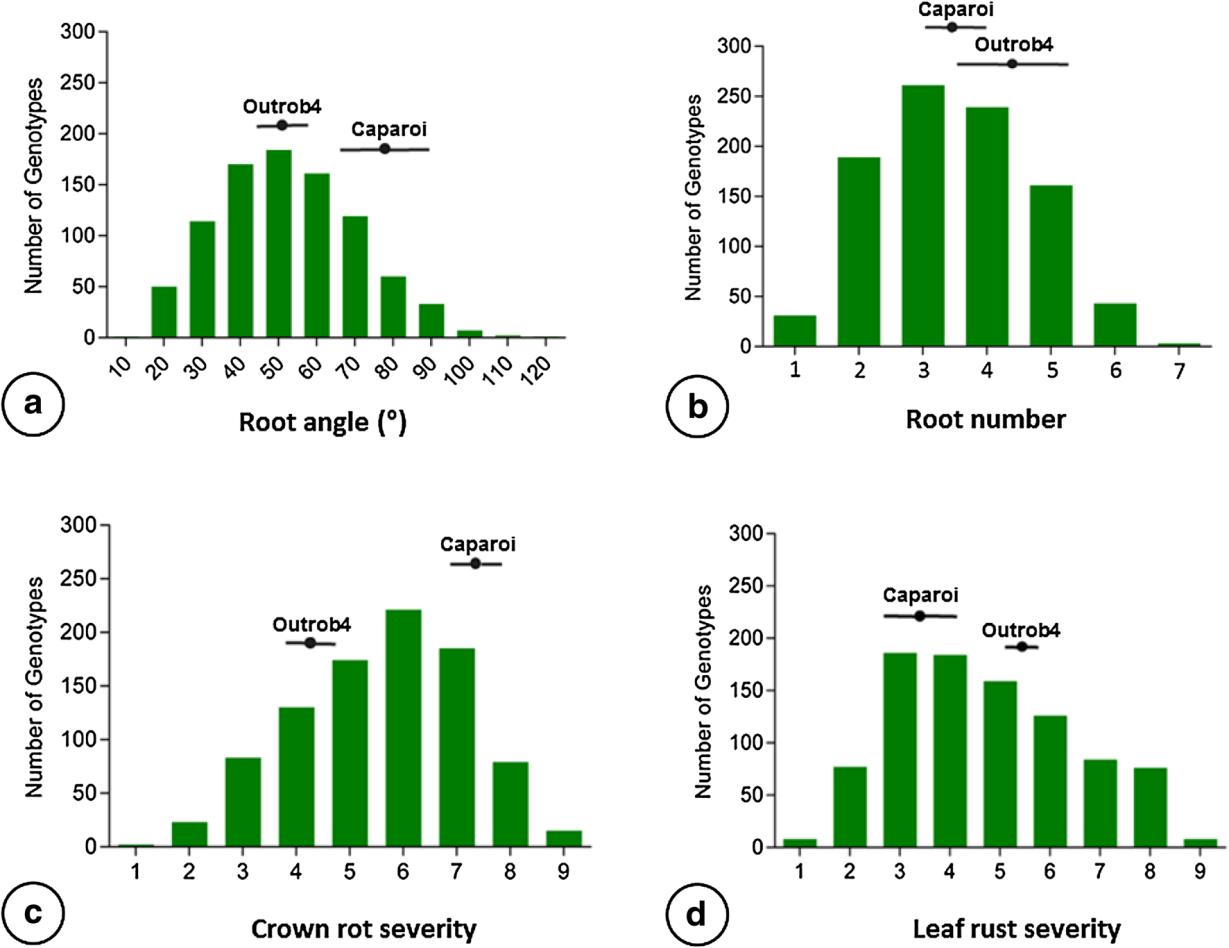
Frequency distribution for the F_2_ segregating population for root angle (**a**), crown rot (**b**), root number (**c**) and leaf rust (**d**). Calculations of parental line means and confidence intervals (95%) are displayed for each trait to highlight the individuals with higher or lower values in comparison to the parents (bi–directional transgressive segregation)

Measures for RA were successfully obtained for 882 F_2_ individuals, as determined by the angle between the first pair of seminal roots. RA could not be measured for the remaining 118 seedlings because one or both roots were not visible. The number of missing values was within the expected range for this method [35]. Outrob4 displayed a narrower RA (50.1°) compared to Caparoi (77.3°). The F_2_ progeny displayed a high degree of variation for RA phenotypes, ranging from 12° to 120° (Fig. 4a). To determine the extent of transgressive segregation, the upper and lower limits of the 95% confidence interval were calculated for both parental lines: Outrob4 ranging 45.1°–54.9° and Caparoi ranging 67.3° to 87.2° (Table 2). Interestingly, the F_2_ population exhibited bi-directional transgressive segregation, where seemingly different sets of genes influencing RA were contributed by both parents. For instance, 38.3% of F_2_ individuals displayed a narrower RA and 3.9% displayed a wider RA when compared to the maximum confidence interval (95%) attained by the parental lines.

**Table 2:** Total number of phenotyped individuals with values for all traits and for each trait separately (root angle, root number, crown rot, leaf rust and plant height) in the F_2_ bi-parental segregating population. Lower and upper values of interval confidence (95%) for each parent were calculated for each trait. Percentage of positive and negative transgressive segregation is displayed for each trait

F_2_ generation	Root angle °	Root number #	Leaf rust Severity 0–9	Crown rot Severity 0–9	Plant height cm
Total number screened	882	927	908	912	916
Outrob4 confidence interval	45.1–54.9	3.4–5.3	5.0–5.7	3.8–4.6	52.36–60.04
Caparoi confidence interval	67.3–87.2	3.1–4.2	2.5–3.7	6.7–8.2	44.9–53.4
Positive transgressive segregants (%)	38.3	4.9	9.3	11.8	20.4
Negative transgressive segregants (%)	3.9	51.8	32.3	10.3	17.2

#### Root number

Measures for RN were obtained for 927 F_2_ individuals. For the remaining 73, RN could not be determined because roots were not visible and in some cases the seed failed to germinate. Images captured 10 days after sowing were used to determine the number of seminal roots. Outrob4 displayed the highest mean RN (4.3) in comparison to Caparoi (3.7). The number of seminal roots per plant varied from one to seven roots (Fig. 4b). Similar to RA, the F_2_ population demonstrated bi-directional transgressive segregation. Individuals deemed to exhibit transgressive segregation were those that displayed phenotypes outside the lower and upper bound (95% confidence interval) of the two respective parental lines (Outrob4 3.4–5.3, Caparoi 3.1–4.2). The number of individuals that showed higher RN than both parents was 45, representing 4.9% of the population.

#### Crown rot response

A total of 912 F_2_ individuals were evaluated for CR during the grain filling stage. Caparoi displayed the most susceptible score amongst the set of parents and standards with an average of 7.5 (Table 1). In contrast, Outrob4 displayed a lower CR mean score of 4.3, which is equivalent to a MRMS response. Confidence intervals (95%) were calculated for CR scores obtained by Outrob4 (3.8–4.6) and Caparoi (6.7–8.2). Based on these limits, the F_2_ population demonstrated bi-directional transgressive segregation: 108 individuals (11.8%) displayed higher levels of tolerance, whereas 94 individuals (10.3%) displayed increased susceptibility to CR (Fig. 4c).

#### Leaf rust response

A total of 908 F_2_ individuals were successfully phenotyped for LR resistance at the adult plant stage. The level of disease intensity and infection was measured on the flag leaf during the early stages of grain fill. A scale of (0–9) was used, where 0 is resistant and 9 is very susceptible. Caparoi displayed MRR response to LR and Outrob4 demonstrated MRMS, as a result, segregation was evident in the F_2_ population (Fig. 4d). Outrob4 obtained a mean score of 5.4 (MRMS), whereas Caparoi displayed a higher level of resistance with a mean of 3.1 (MR). The 95% confidence intervals were calculated for both Outrob4 (5.0–5.8) and Caparoi (2.5–3.7). Falling outside this range, F_2_ individuals were deemed to display transgressive segregation, including 85 that displayed higher levels of resistance, representing 9.4% of the population (Fig. 4d).

#### Plant height

The total number of F_2_ individuals evaluated for PH was 916. PH scores ranged from 24 to 94 cm. Outrob4 was slightly taller (56.2 cm) than Caparoi (49.0 cm). As with all other traits, the F_2_ population demonstrated bi-directional transgressive segregation. The number of individuals significantly shorter than both parents was 187, representing 20.4% of the population while 17.3% of the population displayed taller phenotypes than their parents.

#### Implementing multi-trait selection

**Fig. 5:**
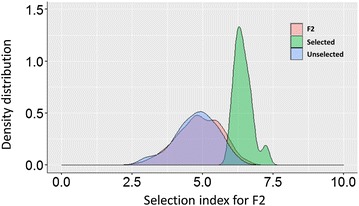
Density distribution of the weighted selection index values for selected, unselected and the entire F_2_ generation (F2). Selection index values are representative of the sum of all traits simultaneously (RN, RA, LR, CR and PH)

Selection of the top 10% of F_2_ individuals using the SI resulted in a mean SI of 6.4 for the ‘selected’ set. Whereas, random selection of 100 F_2_ individuals resulted in a SI mean of 4.78 (Fig. 5). The distribution of SI in this ‘unselected’ set overlapped with the distribution of the entire F_2_ population, thus was considered representative of a truly random population (Fig. 5). The means were also similar: 4.8 for the entire F_2_ population compared to 4.7 for the ‘unselected’ subset of 100 F_2_ individuals.

#### Screening of ‘selected’ and ‘unselected’ F3 individuals

**Fig. 6:**
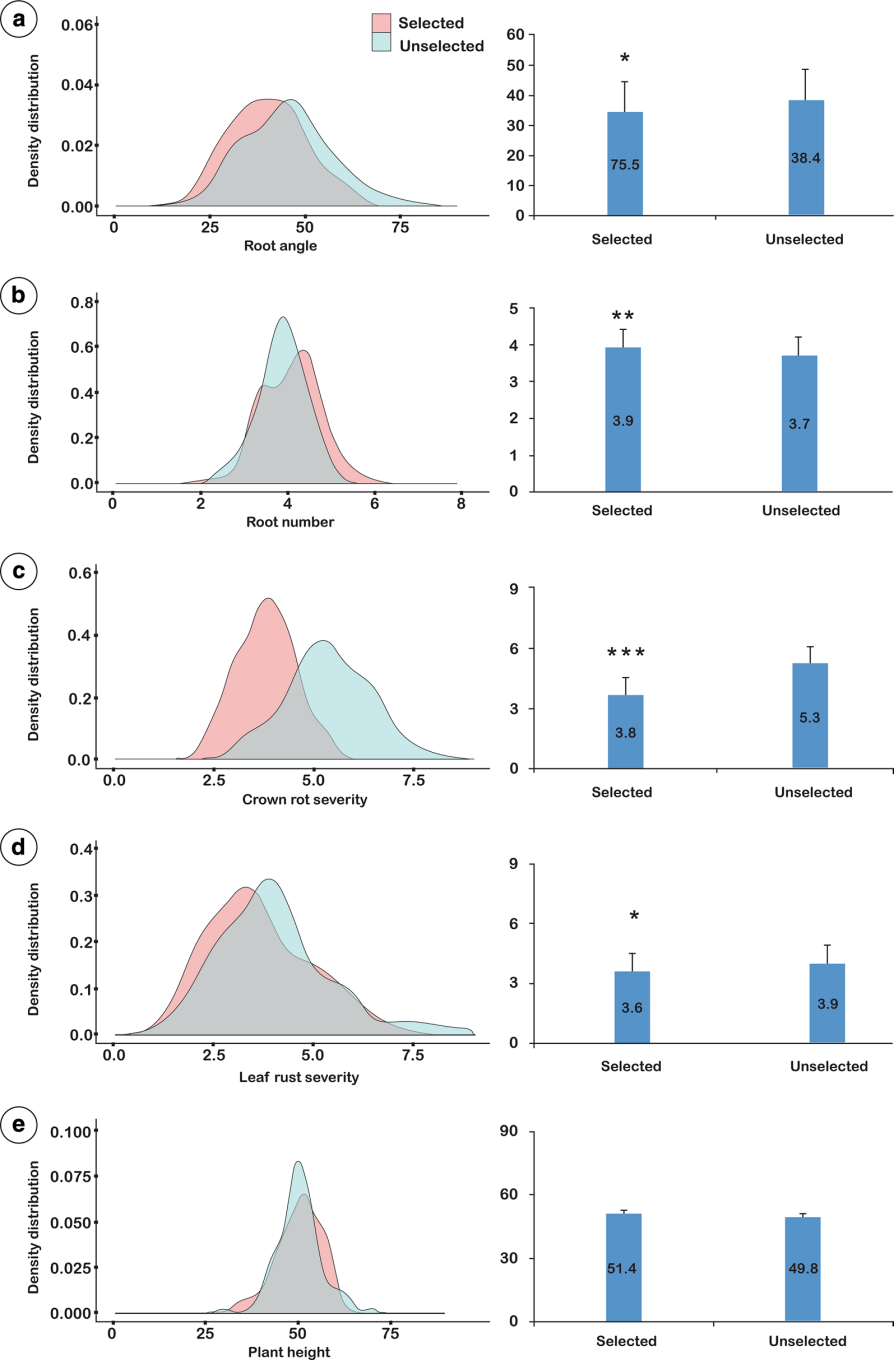
Density distribution and comparison of population means for selected and unselected F_3_ population sets for the following traits: **a** root angle, **b** root number, **c** crown rot severity, **d** leaf rust severity and **e** plant height. Each set includes 100 F_3_ families

A total of 1000 F_3_ plants, plus parents and standards, were evaluated using the multi-trait phenotyping procedure. BLUPs were calculated for all traits (RA, RN, CR, LR and PH) for each of the 100 F_3_ ‘selected’ families and the 100 F_3_ ‘unselected’ (random) families. To highlight the shift in the phenotypes for each trait, the distribution of selected and unselected families are illustrated as density distribution graphs (Fig. 6a–e).

The selected set of F_3_ families displayed a significantly narrower mean RA (34.5°) in comparison to the unselected set (38.4°), representing a significant shift of − 3.9° (*p* value < 0.05). In the F_3_ screening experiment, Outrob4 displayed a very narrow RA (32.4°) in comparison to Caparoi (55.1°). A highly significant shift was also observed for RN, where the mean RN for the selected set was 3.9 compared to 3.7 for the unselected set (p value < 0.01), representing a mean increase of 0.2 roots per plant (Fig. 6b). RN ranged from 2 to7 for the entire population. Outrob4 displayed a higher RN mean (4.7) in comparison to Caparoi (3.5) in the F_3_ screening experiment.

Selection for positive CR response in the F_2_ generation resulted in a highly significant shift toward lower disease severity in the F_3_ selected set (Fig. 6c). Overall, the CR score significantly improved to a MR level (mean score 3.8) compared to the unselected which displayed a MRMS level (mean score 5.3) (p value < 0.001). Outrob4 appeared to contribute the most resistance, displaying a MRMS level of infection (4.7), whereas Caparoi displayed a MSS level (6.8). As expected, the bread wheat standard Sunguard, displayed the lowest levels of infection (mean score 3.8).

Selection for response to LR in the F_2_ generation resulted in a significant shift toward increased resistance in the F_3_ selected set (Fig. 6d). Overall, the mean LR response improved from 3.9 to 3.6. In the F_3_ screening experiment, Caparoi displayed a high level of resistance (2.0) in comparison to Outrob4 (6.4) (Fig. 6d). No significant difference between selected and unselected sets was detected for PH (Fig. 6e). Despite this, Outrob4 displayed a taller phenotype (61.0 cm) in comparison to Caparoi (50.2 cm) in the F_3_ screening experiment.

#### Trait heritability using the multi-trait phenotyping procedure

The broad-sense heritability of the multi-trait phenotyping procedure was calculated for F_2_ and F_3_ experiments. Heritability for the F_2_ experiment was calculated using the replicated parents and standards (inbred lines), as the F_2_ individuals each represent a unique genotype. In the F_2_ screening experiment, the heritability for all traits was high (RA = 0.75, RN = 0.81, CR = 0.78, LR = 0.79 and PH = 0.57). In the F_3_ experiment, using the same technique, the heritability for all traits was also high (RA = 0.62, RN = 0.69, CR = 0.85, LR = 0.79 and PH = 0.82) (Table 3).

**Table 3:** Broad sense heritability calculated for each trait in the F_2_ and F_3_ experiments using inbred lines (parents and standards)

Trait	F_2_ experiment	F_3_ experiment
Root angle	0.75	0.62
Root number	0.81	0.69
Crown rot	0.78	0.85
Leaf rust	0.79	0.91
Plant height	0.57	0.82

## Discussion

Plant breeders are interested in screening a large array of traits in early generations of population development. This enables breeding programs to save time and reduce costs associated with labour and field testing. In order to do so, high-throughput, repeatable and robust screening methodology is required. Improving the existing phenotyping methods and developing novel methods for phenotyping traits are essential for genetic studies and plant breeding. Traits that are highly variable not only in the field but also in the glasshouse require the development of high-throughput, rapid, cost effective and repeatable methods. To meet this need, we developed a screening method to combine multiple traits including root system architecture, LR, CR and PH on the same plant generation, coupled with the rapid generation advancement system ‘speed breeding’.

### Rapid phenotyping fixed lines for multiple traits

The method developed in this study achieved high heritability when screening fixed (inbred) lines. Heritability ranged from 0.57 to 0.91 across experiments for all five studied traits (Table 3). Overall, the heritability of each trait was relatively high and similar to those reported in previous studies [35, 56, 57]. Furthermore, and most importantly, standards included in the experiments displayed similar phenotypic responses under speed breeding conditions to those displayed in the field. For example, Thatcher was used as a susceptible standard and displayed a VS phenotype in both experiments. This result was consistent with those obtained from two previously conducted glasshouse experiments and field trials [38, 58]. Standards for root system architecture included in this study were Mace and Scout. Mace is a widely adopted cultivar and grown on broad acreage around Australia. In particular, this cultivar is preferred by farmers due to its higher yield potential in marginal environments with sporadic rainfall throughout the growing season. Scout by comparison is adapted to the southern regions of Australia with deep soils and known for high water use efficiency and drought adaptation [59]. Seemingly, a narrower and deeper root system would be advantageous in such field environments, while a wider root angle could be more preferred in shallow, sandy soils (for example, in parts of Western Australia). The results for RA standards aligned with the study conducted by Manschadi et al. [60] which revealed that wheat lines grown in deep soils tended to have a narrower root angle and a lower number of seminal roots when compared to wheat lines grown in shallow soils.

The phenotypes displayed by standards in our experiments confirmed the effectiveness of this robust technique for applying selection to RILs and segregating populations. The phenotyping method was designed by integrating two previously reported methods [35, 38]. In the first method, lines were screened for APR to LR adapted to speed breeding and phenotypes were highly correlated to field-based measurements. In the second method, seminal root angle and number were assayed using a transparent pot system, known as the ‘clear pot’ method. This study is novel because it integrated screening of these previously reported traits, plus the CR response, while maintaining high heritability for all traits in a single plant generation. Since the 1980s, scientists have been striving to develop CR screening assays that minimise variation and the time required for infection to occur [14, 49, 61–66]. We achieved this by positioning a piece of agar colonised by *Fp* directly next to the stem of each plant during the tillering stage. This also enhanced the repeatability in F_2_ and F_3_ assays (broad sense heritability ranged 0.78–0.85) and guaranteed infection to take place within 4–5 days. In addition, growing plants under controlled conditions in the speed breeding system not only progressed generations rapidly, but also enabled control of growth conditions to facilitate pathogen development and healthy plant growth. This helps to minimise the variation that can occur in field conditions with a high degree of G × E interaction and high marginal errors when screening individual plants, in the case of F_2_ and F_3_ segregating populations. Nevertheless, it is important to validate levels of disease resistance in the field, as these traits are known for their interaction with environmental conditions and most importantly temperature. For example, the APR gene *Lr34* is considered most effective under low temperatures of 7 °C at seedling stage and less effective at 17 °C and above [67, 68]. Therefore by manipulating temperature, scientists were able to differentiate between Thatcher and Thatcher + *Lr34* at the seedling stage under controlled conditions [69]. Rust infection during the adult growth stage in the field is highly variable and environmentally dependent [38]. The APR gene *Lr34* usually displays a higher level of resistance in the field in comparison to glasshouse conditions [58]. Moreover, variability of CR infection in the field is often due to a combination of factors including temperature, soil surface moisture, stubble residue and inoculum level from previous year [53, 70].

### Multi-trait selection in early segregating generations of durum wheat

Applying selection to F_2_ and F_3_ progenies using the integrated method enabled a shift in phenotypic responses for target traits including CR, RA, RN and LR. The differences that could be observed in the phenotypic responses of the parents resulted in the expression of transgressive segregation in their progenies. This is likely due to the existence of several positive alleles in the parents, which combined via additive or dominant expression into superior progenies. For example, RA for Caparoi and Outrob4 in the F_2_ experiment were 77.3° and 50.1° respectively, while the RA for progenies ranged between 12° and 120°, thus highlighting individuals with narrower or wider RA in comparison to their parents. Transgressive segregation offers an opportunity to apply selection to the individuals with desired combinations of alleles. Selection in the F_3_ focused on retaining individuals with narrower RA, higher RN, tolerance to CR, resistance to LR and shorter PH. That was possible through the use of a weighted SI, with all traits summarised in one single value and the best performers were selected. When the selected set was compared to the unselected set, a significant shift for the mean was noted for all traits (Fig. 5), with the exception of PH. The lack of shift for PH was not entirely unexpected because only low weighting was applied (i.e. 10%) compared to the highest priority traits CR (35%) and RA (30%). Greater gain for the target traits could be achieved by performing multiple cycles of selection.

Selection in early generations is usually effective when applied to populations derived from parents that were phenotypically distinct. Applying selection to early generations of segregating populations is advantageous as it allows enriching the population with desirable alleles [39, 71, 72]. The individuals with undesired combinations of genes are excluded and therefore the cost of field evaluation can be reduced. Despite the variation observed in the F_2_ generation, phenotyping for multiple traits has proven to be an effective tool for excluding unwanted material. Breeding programs routinely apply early generation selection on F_2_ and F_3_ populations using MAS and require robust screening assays to phenotype large numbers of individuals efficiently at less cost. Selection in early generations increases allele frequency of desired traits and therefore the overall efficiency of the breeding program. Selected material in early generations may undertake several testing pipelines before becoming elite material. It is then possible to test elite material in replicated and multi-environmental trials across years which will have more likelihood of success [73].

### Integrating with other breeding tools

MAS is a useful tool to impose additional selection for useful alleles at any stage of the breeding cycle. However, while the cost of genotyping has severely dropped in recent years. The use of MAS is subject to marker availability, which remains one of the biggest challenges. In particular, markers for CR resistance are limited, and all are derived from large QTLs, which lack the resolution to be truly effective [53]. The greenhouse screening method described here is a flexible and deployable system alone, or integrated with MAS if markers are available. The system has the advantage that selection based on phenotype allows identification of individual plants that carry desirable ‘gene combinations’ for the traits of interest. Another strategy could be to screen segregating populations to cull undesirable individuals, which would increase allele frequency of desirable traits in the retained material, prior to conducting MAS. This increases the probability of detecting individuals with all targeted traits using MAS in later generations, making it more cost effective.

The method described in this study has been adapted to speed breeding to enable multi-trait selection in parallel with rapid line development. Speed breeding has reduced the time required to generate RILs with a high degree of homozygosity—only 12 months is required to reach the F_6_ generation. This approach could be combined with GS to identify lines with the highest breeding values for yield or quality, that could be used as parents to further reduce the length of the breeding cycle and increase genetic gain.

## Conclusion

Breeding programs often screen for multiple traits to enhance genetic gain for economically important diseases such as rusts and CR. However, depending on their resources, programs will often lack robust, rapid, high-throughput and repeatable methods for screening multiple complex traits. In this study, we report a novel multi-trait phenotyping method for selecting above and below ground traits, including: root system architecture, LR, CR, and PH. While rapid LR and root system architecture phenotyping protocols were previously reported [35, 38], these techniques were used to characterise fixed lines for a single trait. In contrast, reported here are the separate analyses of these traits integrated with a CR phenotyping procedure, to generate a powerful phenotyping tool for multi-trait selection. Using this method, we applied selection in early generations to enrich the resulting population with desirable allelic combinations for multiple traits. The consistent phenotypes displayed by standards in the phenotyping experiments confirmed the effectiveness of this robust technique for applying selection to segregating populations and shifting the population mean for all target traits simultaneously. PH was the only trait that did not experience a significant shift, as phenotypes were likely impacted by CR infection. This technique is compatible with speed breeding, making it possible to conduct up to four consecutive screens annually, compared to a single screen in the field.

## Additional file

**Additional file 1.** Mean aggressiveness scores for *Fusarium pseudograminearum* isolates collected from farmer fields throughout the norther grain-growing region of Australia. Isolates were evaluated on a susceptible durum wheat cultivar (Jandaroi) and a susceptible barley cultivar (Egypt70) using the 0-9 scale, where 0 indicates host resistance and 9 indicates host susceptibility.
