# Supplementary material for: Speed breeding for multiple quantitative traits in durum wheat
Source: Plant Methods. 2018 May 14;14:36. doi: 10.1186/s13007-018-0302-y (PMC5950182; doi:10.1186/s13007-018-0302-y)
Supplement: Supplementary file 1 — Additional file 1. Mean aggressiveness scores for Fusarium pseudograminearum isolates collected from farmer fields throughout the norther grain-growing region of Australia. Isolates were evaluated on a susceptible durum wheat cultivar (Jandaroi) and a susceptible barley cultivar (Egypt70) using the 0-9 scale, where 0 indicates host resistance and 9 indicates host susceptibility. [file 13007_2018_302_MOESM1_ESM.doc]

**Table S1** Mean aggressiveness scores for *Fusarium pseudograminearum* isolates collected from farmer fields throughout the norther grain-growing region of Australia. Isolates were evaluated on a susceptible durum wheat cultivar (Jandaroi) and a susceptible barley cultivar (Egypt70) using the 0-9 scale, where 0 indicates host resistance and 9 indicates host susceptibility.

| Isolate ID | Mean aggressiveness on  *Triticum durum*  (Jandaroi) | Mean aggressiveness on  *Hordeum vulgare*  (Egypt70) | Overall mean aggressiveness |
| --- | --- | --- | --- |
| Isol_1 | 5.6 | 6.2 | 5.9 |
| Isol_2 | 5.0 | 5.7 | 5.3 |
| Isol_3 | 5.5 | 4.6 | 5.1 |
| Isol_4 | 6.1 | 5.9 | 6.0 |
| Isol_4E | 4.7 | 4.6 | 4.7 |
| Isol_5E | 4.6 | 5.0 | 4.8 |
| Isol_6E | 5.5 | 5.9 | 5.7 |
| Isol_BE | 7.1 | 5.4 | 6.2 |
| Isol_CSIRO | 5.1 | 3.6 | 4.4 |
| Isol_Mixed | 4.4 | 4.5 | 4.5 |
|  |  |  |  |
